# Introducing the Mellorater—The Five Domains Model in a Welfare Monitoring App for Animal Guardians

**DOI:** 10.3390/ani14152172

**Published:** 2024-07-25

**Authors:** Cristina L. Wilkins, Paul D. McGreevy, Suzanne M. Cosh, Cathrynne Henshall, Bidda Jones, Amy D. Lykins, William Billingsley

**Affiliations:** 1 School of Environmental and Rural Science, University of New England, Armidale, NSW 2353, Australia; 2 Sydney School of Veterinary Science, University of Sydney, Sydney, NSW 2006, Australia; paul.mcgreevy@sydney.edu.au (P.D.M.); bidda.jones@sydney.edu.au (B.J.); 3 School of Psychology, The University of New England, Armidale, NSW 2353, Australia; scosh@une.edu.au (S.M.C.); alykins@une.edu.au (A.D.L.); 4 School of Agricultural, Environmental and Veterinary Sciences, Charles Sturt University, Wagga Wagga, NSW 2678, Australia; chenshall@csu.edu.au; 5 Australian Alliance for Animals, 16 Goodhope Street, Paddington, NSW 2021, Australia; 6 School of Science and Technology, University of New England, Armidale, NSW 2353, Australia; wbilling@une.edu.au

**Keywords:** welfare assessment, quality of life, animal husbandry, end-of-life decisions

## Abstract

**Simple Summary:**

This article introduces an animal welfare monitoring app based on the 2020 Five Domains Model that considers how an animal’s nutrition, environment, health, and behavioural interactions, influence their mental state. Adapted for smartphone use, the Mellorater app allows animal guardians (carers, keepers, and owners) to record structured observations of an animal’s life-world with a free research-backed tool. The aim is to help them monitor and improve their animal’s daily lived experiences, make better management decisions, and achieve a good life for their animals. Completing the checklist does not require specialist training and a user-guide with step-by-step instructions on using the app is provided. Users respond to 18 statements by noting their level of agreement with each statement, using a five-point scale from strongly disagree to strongly agree. The authors acknowledge that this form of self-reporting has some limitations but propose that these may be outweighed by the benefits of structured monitoring being repeated over time. This approach helps to identify ongoing shortfalls in an animal’s life-world and trends in their observed quality of life indicators. Both of these outcomes may stimulate contact with sources of further advice, including veterinarians and other animal health and welfare professionals.

**Abstract:**

When monitoring an animal’s welfare, it helps to have comprehensive and day-to-day information about the animal’s life. The goal is to ensure that animal guardians (carers, keepers, and owners) use such information to act in the animals’ best interests. This article introduces the Mellorater, an animal welfare monitoring app based on the 2020 Five Domains Model. This framework provides a means of capturing comprehensive information about the world in which individual animals exist. The Mellorater asks animal guardians to rate their agreement with 18 statements covering any focal animal’s nutrition, environment, health, and behavioural interactions using a five-point Likert scale. No specialist training is required other than following straightforward instructions on using the app, which are provided. The Mellorater is not proposed as a validated welfare auditing tool because it relies on reflective self-reporting and, thus, is vulnerable to the user’s subjectivity. If users’ subjectivity is stable over time, then the longitudinal data may be considered useful proxies for trends in quality of life. That said, it has the potential to be used by trained auditors if scientifically validated, species-specific indicators are applied. The Mellorater collects anonymous data and has been approved for a study to explore how the use of such scales may differ among guardians of different species and in different contexts. In this paper, we conduct the following: (1) summarise the app’s purposes; (2) clarify its capabilities and limitations; and (3) invite animal welfare scholars, veterinarians, health and welfare professionals, and animal guardians to use it.

## 1. Introduction

The past decades have been marked by a progressive shift in people’s attitudes toward animals and, today, there is widespread recognition of animal sentience [1] and the potential for animals to have negative and positive experiences [2,3]. The evolution in social viewpoints has driven three key advances in the field of animal welfare science. First, there has been a notable expansion in both the theoretical and empirical examination of animals’ affective states, resulting in a more comprehensive understanding of how to evaluate them [4,5,6]. Second, the emphasis has shifted from primarily minimising negative experiences to including and promoting opportunities for animals to engage in positive and rewarding experiences [7,8,9]. Third, increased attention has been directed towards the cumulative effects of experiences over time, highlighting the significance of assessing welfare state at multiple given points in time over the course of an animal’s life, also referred to as quality of life [6,10,11]. 

The growing interest in animal welfare has spurred the creation of tools for its evaluation [12,13,14,15]. Nevertheless, there appears to be a tendency to overlook distinctions in whether a tool is meant for assessment, measuring, auditing, or monitoring [16]. To remedy this, we offer the following definitions when using these verbs in this context: Assessment is the process of judging or deciding on the nature and quality of an animal’s state of welfare based on available evidence. Measurement is the process of quantifying the characteristics of an animal’s welfare so it can be compared with a previously established standard. Auditing is an on-site verification activity, such as an inspection or examination, to ensure compliance with established welfare standards and requirements. Finally, monitoring involves observing and checking the progress or quality of welfare over a period, maintaining regular surveillance, and systematically reviewing and reporting on the findings.

A number of industry-focussed animal welfare auditing tools, designed to incentivise welfare improvements through consumer demand, are used internationally. These tools function as on-farm monitoring systems that can translate their results into product information systems and they are typically intended for use by specialist trained auditors [5,12,13,17]. A common aim for such protocols is to measure welfare, i.e., obtain a net welfare outcome by the aggregation of values previously assigned to selected welfare indicators [5]. However, the way in which the scores are assigned and weighted is value-laden and varies depending on the aims of the welfare audit [18], while the process is not always transparent [16]. It has also been argued that numerical tiers can lead people to believe that a high degree of precision is possible when assessing the impact that internal and external conditions have on an animal’s mental state, which is not the case [16,19,20]. Welfare auditing tools that rely on the aggregation of assigned scores are more relevant in large-scale, commercial animal production systems where the aim is to conform with regulatory guidelines or minimum standards. The Five Domains approach cautions against both the assigning of numerical scores and the aggregation of values that reflect negative and positive welfare outcomes. Instead, it recommends the use of separate frameworks for grading welfare compromise and welfare enhancement [6,10,19].

There is a lack of suitable welfare monitoring tools to assist individual animal guardians (carers, keepers, and owners) to assess what is best for their animals [21,22]. Collecting comprehensive and day-to-day information about the factors that affect an animal’s experiences across all domains of welfare and retrieving historic data to identify patterns are essential when appraising trends in their quality of life. We propose that an app that is designed to encourage structured monitoring may help animal guardians make more comprehensive and relevant observations, thereby motivating further monitoring and action based on the data generated. 

The central aim of this type of welfare monitoring tool should be to allow animal guardians to collect and monitor information that can help them, along with veterinary health and welfare professionals, to make informed decisions and to act in an animal’s best interests. For example, to monitor how well the diet is meeting a focal animal’s nutritional needs, a guardian could record changes made to the diet or to how food is presented and score their animal’s body condition. They could then repeat the assessment to evaluate the effect of the changes. This monitoring may require observing the animal directly and on a regular basis, to record any meaningful improvement or decline in condition. If the records kept include all aspects of the animal’s care as well as details of how they interact with their surroundings and other animals (including humans), they may highlight connections between dietary changes and behavioural shifts which might otherwise go unnoticed. 

Comprehensive records are important because some experiences may result from a combination of factors across different domains. One example is exhaustion from a lack of sleep, a condition that is likely to compromise welfare [23,24,25]. In indoor housing systems for cattle or horses, the provision of suitable bedding substrate is commonly assumed to lead to a better welfare outcome; however, there is a relationship between recumbency and welfare state that may be independent of bedding. For example, musculoskeletal injuries can affect an animal’s ability to lie down and, afterwards, get to their feet again, such that the affected animals are less likely to sleep normally despite being on a suitable substrate [24]. Other factors, such as available lying space and social interactions, can also affect sleep patterns [26] and may vary as the animal ages [27]. 

These examples highlight that evaluating and monitoring animal welfare requires a systems-thinking approach. Recording what an animal is doing during a 24-h period, together with a broad range of welfare-relevant aspects of the animal’s life, can provide invaluable insights into the welfare state of the animal and the conditions that are contributing to it. Graphing the data from two or more observations should allow animal guardians to appreciate trends and assess them against the threshold of acceptable quality of life that they and other carers (including veterinary health professionals) have agreed upon in advance. The keeping of complete records that capture data about a broad range of the animal’s experiences will facilitate future management decisions that can improve the animal’s quality of life. 

The Mellorater is based on the 2020 Five Domains Model for Animal Welfare Assessment and Monitoring. The software was developed to provide animal guardians with a free app featuring a complete but easy-to-use checklist to help them as follows: (a) monitor their animal’s life and welfare; (b) make better management decisions affecting their animal; and (c) achieve a good life for their animal. This article explains how the Mellorater app was developed and is intended to be used in animal care and research. 

### 1.1. The Five Domains Model

**Figure 1:**
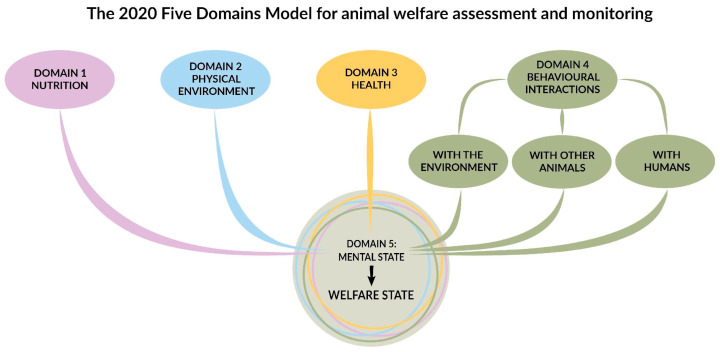
The 2020 Five Domains Model for Animal Welfare Assessment and Monitoring. By Cristina Wilkins adapted from Mellor et al., 2020 [10].

An animal welfare framework that is being adopted across all animal sectors is the Five Domains Model for Welfare Assessment and Monitoring [10]. The Five Domains Model aligns with current scientific understanding indicating that it is possible to identify if an animal’s internal physical and functional states and their external circumstances are giving rise to negative and/or positive subjective experiences or affects. Negative emotions are linked to compromised welfare, while positive emotions are associated with enhanced welfare [4,20,28]. The model was originally proposed in 1994 [29], and then revised in 2001 [30], 2004 [31], 2009 [32], 2012 [2], 2015 [33], and 2020 [10]. It is valuable for assessing animal welfare because it builds on the increasing neuroscientific understanding of the brain processes that underlie aversive or negative and rewarding or positive affects and their physiological and behavioural manifestations [4,20,34,35,36,37,38]. The current version of the model highlights five key domains: Domain 1, nutrition and hydration; Domain 2, the physical environment; Domain 3, health and fitness; Domain 4, behavioural interactions; and Domain 5, mental state. This model underscores animals’ agency-related interactions with their environment and other animals, including humans, by renaming Domain 4 which was previously “behaviour” in the previous versions, to “behavioural interactions” (see Figure 1). 

The aim of each of the five domains is to draw attention to all the areas that are relevant to how animals experience their life-world, while recognising the unique and evolved sensory abilities of their species. Classifying the extensive array of experiences according to specific domains allows for model-based welfare assessments to be structured, systematic, comprehensive, and coherent. Engaging in this process directs attention to general areas of welfare concern, helps identify their likely sources, and adds granularity to experiences that are often overlooked or generalised into broader, less specific descriptors. For example, a Five Domains-based evaluation of ‘discomfort’ within Domain 2, the physical environment, prompts a determination of whether the discomfort is auditory, thermal, visual, olfactory or physical, which enables more precise management changes and monitoring of responses. The approach facilitates a qualitative grading of specific experiences according to the severity of functional impact, related intensity, and duration, and whether or not these impacts need to be urgently mitigated. 

The Five Domains Model has been used to assess welfare risks and opportunities for enhancement prospectively and retrospectively, allowing decision makers to select new approaches to management and handling in a wide range of species [19,32,39,40,41]. Such use of the model also makes available information that is invaluable for informing end-of-life decisions [20].

### 1.2. Development of the Mellorater Checklist

In 2018, a checklist was created by BJ and PM, consisting of a series of 20 statements relating to the Mellor & Beausoleil (2015) version of the Five Domains Model [33]. Its aim was to help undergraduate students enrolled in Understanding Animal Welfare, an open learning course offered by the University of Sydney, to conduct an animal welfare assessment exercise. In 2021, PM and CW proposed the use of the same checklist in making end-of-life decisions for horses [42]. The initial Mellorater checklist was developed subsequently to align the 2018 template with the latest version of the Five Domains Model. It now consists of 18 statements, three for each of Domain 1, Domain 2, and Domain 3, and three for each of the behavioural interactions subdomains in Domain 4. This checklist forms the basis of an open learning online short course on applying the Five Domains within the context of sport and recreation horses [43]. The course is approved as a provider of continuing education by the American Veterinary Boards Association, the New Zealand Veterinary Association, and the British Horse Society, among others. 

Designed for use in all species in captive or managed environments, the checklist has now transitioned from a paper-based template to an online platform, facilitating the collection and storage of data over time to help animal guardians and researchers record and visualise trends. Data from the app are being gathered as part of an approved study to identify variance in respondents’ use of the Likert scales provided and how these outcomes differ across species (in the first case, horse guardians versus dog guardians), as well as within the same species in different contexts. Importantly, one of the chief merits of this framework is that it allows structured discussion between animal guardians and healthcare professionals.

The Mellorater app is now licensed under Creative Commons Attribution-Non Commercial-No Derivatives, and will be made accessible at no cost to users, thus enabling broader access. 

### 1.3. How the Mellorater App Works

**Figure 2:**
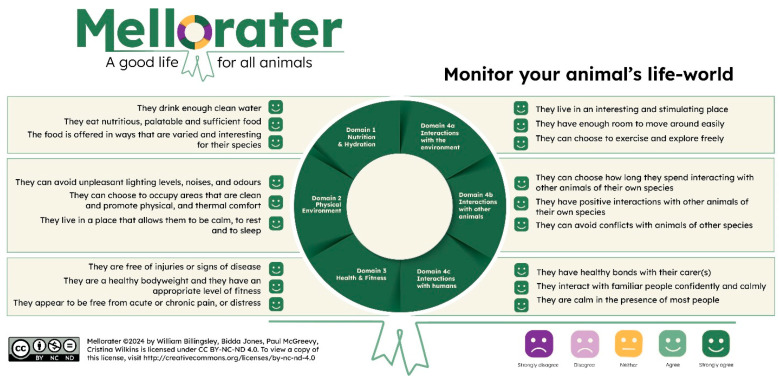
The 18 statements in the current version of the Mellorater and how they relate to the four physical domains: nutrition (and hydration); physical environment; health (and fitness); and behavioural interactions with the environment, other animals, and humans. Illustration by Cristina Wilkins.

The Mellorater app uses a checklist of 18 statements based on the 2020 Five Domains Model for animal welfare assessment and monitoring (Figure 2). The checklist encourages users to observe a focal animal they are assessing and the conditions they are kept in. It then asks them to consider their level of agreement with each of the 18 statements. In addition, users can save notes and upload images for future reference. Given that it is primarily an attention-focusing and reflection tool, the user guide encourages guardians to record information and make decisions that are in the animal’s best interests. Users are also prompted to log their level of confidence in the evidence they used to determine their response to each statement. Acknowledging uncertainty when evaluating welfare is beneficial, particularly since users will be making qualitative evaluations [19]. The intention is to prompt the user to question the reliability and quality of the evidence used, and to seek expert advice when needed. 

**Figure 3:**
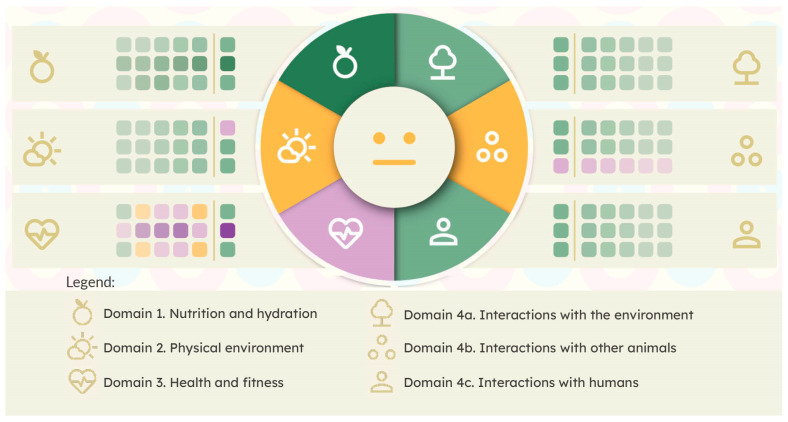
A compact aggregated visualisation of recent assessments for an animal. The central dial summarises the most recent assessment, with one segment per domain. Domains 1 to 3 appear on the left of the figure and Domains 4a, 4b, and 4c on the right. The responses to the three individual statements in each domain are shown (as coloured rectangles). Each column of the grid represents an assessment. The most recent assessment is in the middle (closest to the summary dial), with past assessments radiating outwards and faded with time. The colour scale uses purple for low values, orange for medium values, and green for high values; this colour scheme is accessible to people who are colour blind. It also avoids the use of red which may be considered alarming.

Once completed, the software displays the overall results in a summary card, providing a compact and at-a-glance outline of the animal’s status (see Figure 3). The centre dial summarises the most recent survey, with a segment for each domain, colour-coded by a heuristic that considers whether any statements within the domain were lower-rated. After repeated uses, the longitudinal trends in the responses to the statements are displayed alongside the summary card to give a visual indication of reported improvement or deterioration of the animal’s conditions. For example, in Figure 3, the domain on the east of the dial is interactions with other animals; this animal’s guardian reported a high level of agreement with the first two statements but consistently disagreed with the third statement (whether it can avoid conflict with animals of other species). In the physical environment domain (west), the animal’s wellbeing was scored highly except that in the most recent assessment, it was scored low on the first question (whether the animal can avoid unpleasant lighting levels, noises, and odours). (More information on how the software manages the results can be found in the Technical Details Section below). 

As explained in the introduction, the relative impact of the different domains on welfare is unknown and is likely to differ across species. Thus, the summary emphasises the hotspots for attention in specific domains of the animal’s world instead of scoring an absolute value of good or poor welfare. Overall, the aim is to encourage reflection by the individual user, first on the quality and reliability of the evidence of a poor or high welfare outcome, and second on the appropriate next step, which could be to act, look for more evidence, or seek expert advice. For example, when a user records a high level of confidence and a low welfare outcome, the summary card will prompt them into action. In contrast, a low level of confidence combined with a low welfare outcome will encourage the user to find more evidence or seek expert advice. 

Individuals and teams can use the app in various ways to optimise shared monitoring of the same animal(s) over time. For example, they could follow a series of steps to make their assessment repeatedly, over days, to plot quality-of-life trends. Similarly, welfare organisations charged with rehabilitating and rehoming animals could use it to monitor changes, keep records of the animals they manage, and monitor their transition to new homes. The benefit of using digital records, rather than hard copy, is that they are stored securely and can be shared immediately among relevant, authorised stakeholders. The flexibility of online record-keeping is illustrated by the example we offer in Table 1 of steps that users of the Mellorater may wish to take when monitoring focal animals.

The contexts in which animals’ life-worlds are observed are diverse and dynamic. For example, a companion dog’s experiences when they are home alone will differ when their human carers are with them, or when they are in the dog park. Similarly, in a zoo, the animals’ experiences in the exhibit and off-exhibit will differ. To account for this, the app provides guardians with the opportunity to log the date and situation under which data are recorded; for example, they can record that their dog is at home without carers for 5 days/week for more than 2 h/day, or that their horse is turned out for 8 h/day 5 days/week. The app offers a series of drop-down menus for common contexts and indicative durations and frequencies of these situations for a focal animal. Currently these are offered only for dogs, horses, and cats. 

### 1.4. User Experience

While many guardians will routinely consider the welfare of animals in their care, most will not be accustomed to completing structured, systematic welfare evaluations across all the domains. It is possible that some users may be disturbed by receiving feedback that indicates potential shortcomings in the care they are providing to their animals [44,45,46,47,48,49,50]. For this reason, in the app information sheet and enrolment guide, we ask whether users are willing to accept the risk of receiving a negative result. All users are advised that, if the results raise concerns, they should discuss their animal’s welfare with a qualified professional such as their veterinarian. 

Additionally, they are advised that data are being collected as part of an ethics committee approved study, and that in reporting the development of the app, the research team may wish to quote users’ qualitative feedback but that any such quotes will be anonymous. Data collected using the Mellorater are gathered under the approval of the University of New England Human Research Ethics Committee (Approval number HE22-136). 

## 2. Technical Details

The app uses a JavaScript user interface framework that runs within a web browser. This allows for a progressive deployment strategy, as well as movement between desktop and mobile use. During user testing and early use, the app is accessed via a URL that can be pinned to a smartphone’s home screen, allowing a rapid update and deployment cycle as new uses are identified and interface issues are fine-tuned. As the app’s use broadens, it will be adapted to enable users to install the app onto their smartphones as a “Progressive Web App” for offline use. A version may also be released via app stores at a later date.

The app visualises, summarises, and helps users to explore the data collected from animal assessments over time. With eighteen statements per assessment, and the need to see trends for each animal at a glance, visualising this information in the compact space of a smartphone screen is a central design challenge. It is also one where the appropriate solution may be expected to evolve as the user community becomes more familiar with monitoring animals’ life-worlds, and as the data about how people assess the welfare of animals in practice improve. Consequently, the app is built using a user interface toolkit, originally designed for education, that allows rapid development of interactive visualisations [51].

Each visualisation is designed to balance the need to summarise data for usability reasons, with a requirement to ensure that any aggregation heuristics do not hide data or override human judgement. Figure 3 shows a summary card—a particularly compact visualisation shown for each animal on the app’s home screen. This needs to show an at-a-glance summary of the animal’s status, including trends in its wellbeing over time. To make this legible on a small smartphone screen, the central dial summarises the most recent survey, with a segment for each domain, colour-coded by a heuristic that considers whether any statements within the domain were lower-rated. However, to ensure this visual heuristic does not hide lower-level detail, the trends in the animal’s welfare are visualised at the statement-level beside the dial. Other screens within the app have other purposes, such as exploring assessments in detail, including any notes taken, but in each case the concept that data aggregation must not become data hiding is a central principle of the design.

## 3. Discussion

### 3.1. How the Mellorater Can Help Animals

Completing the Mellorater will prompt animal carers to reflect on aspects of the animal’s life-world that are known to interact with and influence physical function and mental state. Each statement redirects attention away from the evaluator’s perspective to that of the animal, reminding users of the wide range of conditions that matter to animals in terms of their welfare. The 2020 Five Domains Model provides the scaffolding and scientific basis to ensure that animal carers do not overlook aspects of the animal’s life-world that science has shown to be important to them. 

Humans are inclined to overlook inconvenient truths [52,53,54,55,56]. Studies have shown that animal sector stakeholders tend to prioritise the tangible aspects of welfare and the resources that are provided to animals, and may disregard the animal’s affective experience [47,48,57]. By ensuring users are addressing three physical domains alongside three categories of behavioural interactions, the Mellorater aims to overcome these psychological limitations. The app aims to stimulate animal guardians to improve the lived experience of individual animals incrementally. It does this by highlighting specific areas that align both with good welfare outcomes and hotspots for improvement. The purpose of the app is to permit collection of online data files to record how any animal’s current welfare may be compromised, maintained, or enhanced. It is designed to allow guardians to share data on focal animals, regardless of the current caregiver. In identifying the opportunities to improve an animal’s life-world, the read-out from the app provides recommendations for improvement and identifies appropriate sources of further information on a given domain (e.g., by referring users to RSPCA Australia’s Knowledgebase). 

The data storage capacity facilitates graphing of trends in the welfare status of an animal over time and may also assist in making ethical management decisions. For example, there are instances where certain restrictions are imposed in an animal’s best interest, either temporarily or to mitigate other risks. One example may be post-surgery restrictions imposed on a cat who is confined to a small crate, must wear an Elizabethan collar to protect them from further injury, and have their access to food and water restricted during the recovery period. Another example may be of a dog with severe fear-induced aggression towards strangers, who has their living environment and interactions with humans restricted to mitigate other risks. A third example may be of a pony with metabolic dysfunction who is either confined to a dry lot or wears a grazing muzzle to reduce the risk of painful laminitis. In these cases, and consistent with the Five Domains Model approach, low welfare readings are to be expected in certain domains, even if the restrictions are imposed in the animal’s best interest. It is anticipated that, while the animal’s guardian will have straightforward awareness of the limiting factors, there may be a need to record such instances. To this effect, the Notes fields within the app allow users to record specific and local circumstances as memory aids and to help explain why a low welfare reading in any domain is to be expected. The app’s visual representation of longitudinal trends (Figure 3) will highlight the duration of such restrictions, while the colour coding maintains the guardians’ awareness that these conditions are generally associated with negative mental states. This may help those who wish to identify thresholds beyond which an animal should be euthanased or at least not be maintained in the current environment. 

### 3.2. How the Mellorater Can Help Animal Guardians

Animal welfare is complex and dynamic, hence comprehensive assessments require a systems-thinking approach [58]. Systems thinking is recognised as an essential skill in the health sciences because when a patient is sick, diagnosing the problem tends to lead to better outcomes than treating their symptoms one at a time [59,60]. It is now widely accepted that physical health has psychosocial and welfare impacts. A systems-thinking approach facilitates the delivery of more comprehensive healthcare needs at varying levels and in ways that respect the patient’s perspective [59]. There is a growing consensus among education experts that teaching systems thinking is essential across all levels of education [60,61,62,63]. This is because it helps people understand complex and dynamic systems across multiple contexts, including environmental issues, physical and social systems, and science education in general [64,65,66]. A single intervention can enhance comprehension of fundamental concepts in systems thinking. Monroe and colleagues investigated the learning outcomes of a brief systems-thinking intervention at the undergraduate level; they found that practical application models were better in motivating students to challenge and adapt pre-existing beliefs [67]. The Five Domains approach to animal welfare assessment and monitoring corresponds with systems thinking because it considers the complex interrelations among conditions, physical state, and mental experiences across different domains [58]. Fletcher and colleagues demonstrated that a straightforward infographic based on the Five Domains model enhanced horse owners’ understanding of animals’ subjective experiences and welfare-influencing factors. However, they also revealed a demand for accessible and engaging welfare assessment tools [22]. 

The Mellorater app, being based on the Five Domains Model, supports users in adopting a systems-thinking approach by helping them visualise the complexities and dynamism of animal welfare. Through engagement with the app, it is anticipated that users will gain insight into the interconnectedness of the welfare domains, thus enhancing their problem-solving skills.

### 3.3. How the Mellorater Could Direct Animal Welfare Science in the Future

The app collects anonymous data on animals’ life-worlds but also on how they are reported by the guardians of different species and the same species in different contexts. The primary purpose of the approved research is to reveal any variability in the responses to the 18 fundamental animal care questions that is associated with the species of animal being reported. The data collected are vulnerable to user subjectivity, however, they are not intended for assessing the app’s validity as a welfare measuring tool. It is well-established that self-report questionnaires can provide invaluable insights into how people conceptualise complex topics, such as animal welfare and management. Mellorater intends to capture the experiences and perceptions of those closest to the animals regardless of their level of expertise. Self-reporting is a well-established research tool, often used in citizen science, which allows individuals to contribute valuable data and insights. The robustness of Mellorater data will increase with the quantity collected and the number of species assessed. Data will, for example, show how areas for improvement in animal welfare are distributed differently according to the animal species and the context and purpose for which they are being kept. We are also interested in understanding how and whether the subjectivity changes with repeated use; for example, whether use of the confidence measure becomes more sophisticated. If there is evidence of species-dependent difference in the way scales are used, these can be reviewed to correct species-specific biases, thereby refining the accuracy of the results over time. 

Additionally, the Mellorater’s easy-to-use platform for collecting, storing, analysing, and applying welfare monitoring data related to the Five Domains of welfare could advance animal welfare science in a variety of ways; for example, as follows:

i. To screen animals used for scientific experiments. Researchers could use the Mellorater to evaluate and monitor the suitability, health and welfare of animals in experiments by characterising how their welfare is being managed across all domains. Adopting the Mellorater checklist provides a standardised approach that may help ensure that data collected in research contexts are comparable, thus potentially improving the repeatability of studies.

ii. To study the pre- and post-effects of interventions. Use of a consistent welfare monitoring checklist could support researchers in determining how education, management, and husbandry interventions affect a focal animal’s welfare across all five domains. 

iii. Post-intervention assessments and welfare monitoring. The Mellorater can be used to monitor and record the welfare consequences of veterinary and behaviour interventions across all domains, whether the checklist is completed at home by the animal’s guardian or by expert consultants and veterinary staff in follow-up visits. For example, while users may need their veterinarian’s help to assess and monitor their animal’s health domain, the other physical domains that, along with health, feed into the fifth (mental) domain (i.e., interactions, nutrition, and environment), rely on input from users’ own observations and are all relevant when evaluating the animal’s quality of life trajectory. Owners may wish to discuss with their veterinarian or behaviour consultant which outward signs (e.g., evidence of reduced appetite and pain, reluctance to interact) as well as what types and duration of behavioural restrictions are critical points from which to draw data that should be factored into making end-of-life decisions, as well as management decisions, to improve the welfare of animals we do not intend to euthanise.

iv. To assist in making end-of-life decisions. Repeated use of the Mellorater may reveal an animal’s quality of life trajectory and allow carers and veterinary teams to anticipate and set thresholds beyond which euthanasia becomes a priority. This could be especially useful for animal holding facilities such as rescue shelters, laboratories, and zoological collections, because as well as preventing delayed euthanasia, data on quality of life can help prevent trauma or stress in animal guardians during euthanasia by providing a clear rationale for their actions [68]. 

v. For day-to-day monitoring of the welfare of animals held in homes, laboratories, and zoological collections that require the keeping of detailed records. Mellorater data could ensure that animals’ welfare monitoring records are comprehensive and meet regulatory requirements such as those imposed by animal research ethics committees. This requirement is mandatory in some countries [69]. The well-organised methodology of the Mellorater may help users explain to others how they are monitoring and managing their animal’s welfare in a detailed way. As such, it does not provide proof of an animal’s welfare state. 

vi. For educational interventions. The Mellorater can be used as a training tool by animal welfare educators and attending veterinarians; for example, to conduct mock animal welfare assessments during the induction or retraining of animal guardians, as well as to teach them to identify and apply resource-based and animal-based welfare indicators that are relevant to the species being assessed. 

## 4. Limitations

Critics of the Five Domains approach warn of its “potential for manipulation”, and have proposed that it (a) lacks clear principles for any aggregation of welfare measures, and (b) would benefit from repeatability [16]. Nevertheless, they concede that the Five Domains Model is useful for systematic consideration of all sources of possible welfare compromise and opportunities for enhancement. These are the proximate goals of the current software. Ultimately, it is anticipated that feedback from users of the Mellorater will reveal species-specific differences in observations. These data will facilitate refinements that ensure that any species-dependent tendencies of guardians will be reflected in weightings assigned to each of the statements that owners are asked to reflect upon.

**Figure 4:**
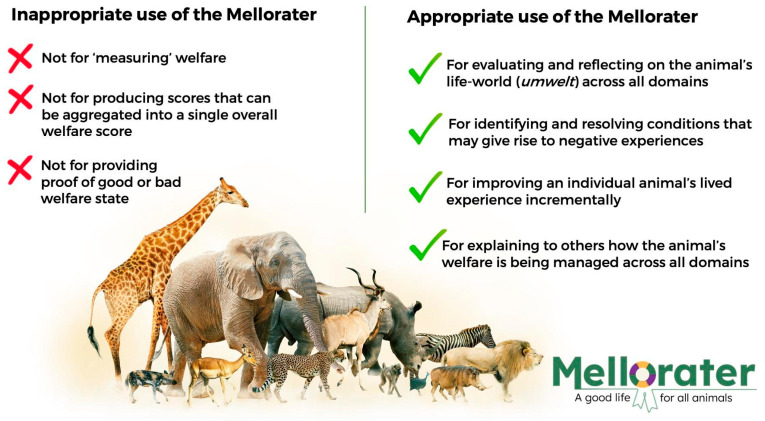
A guide summarising how to use the Mellorater app and limitations on what it offers.

Figure 4 illustrates the output of the Mellorater app on what it offers. The Mellorater is not a welfare measuring tool. It relies on self-reporting and, as such, it is vulnerable to user subjectivity. The statements do not cover all the possible parameters that play a role in determining the welfare state of an individual animal. Nevertheless, and consistent with the foundational guidelines of the Five Domains Model, the checklist covers a broad range of aspects that are known to influence animal welfare outcomes. Thus, it serves as a memory aid and attention-focusing device that prompts users to reflect on the physical and behavioural domains of their animal’s life-world. Importantly, it does not set thresholds of what is or is not acceptable welfare. Instead, being species-agnostic, it is designed to help the user identify husbandry practices that are commonly associated with positive mental states in animals, so they can be maintained, as well as to highlight areas of potential compromises to the animal’s welfare. Repeated use will highlight the duration of such compromises and enhancements, and stimulate further inquiry, including the seeking of professional health and welfare advice. 

The app is not designed to generate scores that can be aggregated into a single overall welfare score. The primary reason for this is that the welfare impact of experiences in the different domains is not known and differs with each species because of the diverse evolutionary histories. We cannot justify attempts to apply scores to each of the four physical domains, and app users are advised to be careful not to assume that each domain is as equally impactful on their animal’s quality of life as the next. 

## 5. Conclusions

The Mellorater app structures record-keeping on the physical domains in which animals live and how they interact with their surroundings and other animals, including humans. It is designed for use in all species in captive or managed environments. The app’s aim is to facilitate the collection and storage of data to assist animal guardians monitor and improve their animal’s life and welfare, to help them make better management decisions and achieve a good life for their animal(s). Although completing the checklist does not require specialist training, the Mellorater has the potential to be used by specialist auditors. This may be achieved by selecting and applying species-specific validated indicators to rate the level of agreement with each of the 18 statements. Importantly, it is not proposed as a validated welfare auditing tool because it relies on self-reporting and, as such, is vulnerable to the user’s subjectivity. Nonetheless, if the user’s subjective perspective remains stable over time, the longitudinal data produced by the app may provide a useful representation of quality-of-life trends. As such, this app may assist animal guardians monitor their animal’s lived experiences as well as aid in end-of-life decision-making for animals. 

A preview of the Mellorater app, which includes a user demonstration, is available online and can be accessed on this link: https://www.mellorater.org/contact (accessed on 20 July 2024).

## Figures and Tables

**Table 1:** Suggested steps that users of the Mellorater can take when monitoring focal animals.

1. To complete an assessment, consider, one-at-a-time, the 18 statements and decide on your level of agreement with each using the 5-point Likert scale. 2. Consider and record your level of confidence in the quality of the evidence you used to agree or disagree with the statements. 3. If necessary, use the notes and/or image feature to save additional records. 4. Once you have completed the checklist, submit your answers to view the summary card and feedback. 5. Identify any statements with which you strongly disagree, as these are the most urgent areas for improvement. If you strongly disagree with a high level of confidence, act without delay to improve your animal’s welfare. If you have a low level of confidence, try to collect more reliable evidence and/or seek professional advice to do so, at the earliest opportunity. 6. For all the statements where there is room for improvement, reflect on what changes could be made to move your decision to Strongly Agree. If you have a high level of confidence, take action. If you recorded a low confidence level, collect more evidence and/or seek expert advice. However small, incremental improvements can make a meaningful difference to your animal’s daily lived experience. 7. Finally, reflect on the statements you strongly agree with. These are aspects of your animal’s life-world that align with good welfare and should be maintained. 8. Repeat the process over several days or weeks to plot your animal’s welfare state over time. The app will produce a graph to help you observe trends in quality of life over time. 9. Discuss the results with your veterinary professionals if you are concerned by a single assessment or trends over time, and seek expert advice if you are not sure how to achieve an improvement. 10. To reduce the subjectivity of your assessment, we suggest you invite other users to conduct their own assessment on the same animal, using these instructions. Consider any discrepancies in the results between observers, discuss the evidence used in your decisions, and see if you can agree to moderate those different assessments. The aim of such discussions should be to find opportunities to enhance your animal’s life-world.

## Data Availability

The data presented in this study are available on request from the corresponding author.
